# Acute intraperitoneal lipopolysaccharide influences the immune system in the absence of gut dysbiosis

**DOI:** 10.14814/phy2.13639

**Published:** 2018-03-13

**Authors:** Kristyn E. Sylvia, Gregory E. Demas

**Affiliations:** ^1^ Department of Biology Indiana University Bloomington Indiana; ^2^ Center for the Integrative Study of Animal Behavior Indiana University Bloomington Indiana; ^3^ Program in Neuroscience Indiana University Bloomington Indiana

**Keywords:** Gut microbiome, immune system, lipopolysaccharide

## Abstract

There is bidirectional communication between the immune system and the gut microbiome, however the precise mechanisms regulating this crosstalk are not well understood. Microbial‐associated molecular patterns (MAMPs) within the gut, including lipopolysaccharide (LPS) that produces a quick and robust activation of the immune system, may be one way by which these interactions occur. Endogenous levels of LPS in the gut are low enough that they do not usually cause disease, although, in times of increased LPS loads, they may be capable of increasing vulnerability of the gut to pathogenic bacteria. Furthermore, chronic, low‐grade inflammation can have lasting effects on the gut, but the effects of acute inflammation on gut communities have not been thoroughly assessed. In this study, we first investigated whether a single modest dose of LPS administered to adult male and female Siberian hamsters (*Phodopus sungorus*) activated the immune system by measuring levels of circulating cortisol and the proinflammatory cytokine TNF‐*α* in the liver compared with saline‐treated animals. We then investigated whether this same acute dose of LPS altered the microbiome 48 h after treatment. We found that, although LPS increased cortisol and liver cytokine levels, and produced changes in food intake and body mass in both sexes, immunological changes were independent of gut dysbiosis 48 h after LPS injection. These data suggest that an acute immune activation may not be capable of altering the gut microbiome in healthy individuals. It is likely, however, that this type of immune challenge may have other physiological impacts on the gut's vulnerability, and future studies will investigate these relationships further.

## Introduction

The immune system, the hypothalamo‐pituitary‐adrenal (HPA) endocrine axis, and the microbiome interact during times of both health and sickness; however, the potential points at which crosstalk among these systems takes place are not completely understood. The microbiome is not only a diverse, host‐specific, and symbiotic bacterial environment, but it also contains its own immune response system, which integrates information from inside and outside the gut (Powell and MacDonald 2017). Interestingly, germ‐free (GF) mice that lack normal gut microbiota exhibit altered development of many aspects of the immune system, which prohibit the immune system from responding appropriately (Hoshi et al. 1992). Furthermore, GF mice do not exhibit the same behavioral response to an immune challenge as seen in wild‐type mice (e.g., lethargy, decreased food intake), indicating that the microbiome may play a large role in the behavioral responses seen during sickness (Campos et al. 2016). Moreover, certain types of bacteria may be important for immunomodulation, and mounting an appropriate immune response may help mediate the effects of pathogenic bacteria in the gut (Dinan and Cryan 2012).

Treatment with lipopolysaccharide (LPS), a cell wall component of gram‐negative bacteria, is commonly employed to induce an immune response in animals, triggering increases in circulating corticosterone and proinflammatory cytokines (Bilbo and Schwarz 2012; French et al. 2013). The use of LPS mimics a bacterial infection by binding to the toll‐like receptor (TLR)‐4, which contributes to the sickness response (Harvey and Boksa 2012). Furthermore, in times of immune challenge, neurons from the hypothalamus, pituitary, and adrenals participate in the HPA response, releasing corticotropin‐releasing hormone (CRH), peripheral release of adrenocorticotropic hormone (ACTH) and downstream glucocorticoids (e.g., cortisol, corticosterone) to act on target tissues (Lowry 2002; Viau 2002; Zhang et al. 2002; Herman et al. 2005). Interestingly, gut dysbiosis, characterized here as a disruption of gut microbial communities, is associated with abnormalities in the HPA axis (reviewed in Mulle et al. 2013). For example, plasma ACTH and corticosterone are elevated in response to restraint stress in GF mice when compared with control mice (Sudo et al. 2004), suggesting the HPA axis plays a likely role in many of the behavioral consequences of dysbiosis.

Endogenous levels of LPS present in the gut are sufficiently low that they do not usually cause disease; however, in times of elevated LPS loads, they may be capable of increasing permeability of the gut lining and thereby influencing vulnerability of the gut to pathogenic bacteria (Cani et al. 2007a,b). For example, in times of chronic stress, the gut epithelial layer will become more permeable. This increased permeability can lead to increased movement of endotoxins from inside the gut to the external systemic system, which can create low‐grade inflammation for long periods of time (De Punder and Pruimboom 2015). Importantly, acute stress also increases intestinal permeability. In particular, 4 h of either restraint or cold stress significantly increases the secretion of chloride ions out of the gut and into circulation (Saunders et al. 1994), suggesting that this short‐term stressor is capable of greatly influencing the lining of the gut and producing important downstream effects. Furthermore, work has suggested that TNF‐*α* plays a particularly important role in maintaining proper gut permeability. For example, the inhibition of TNF‐*α* reduces the permeability of tissues in the small intestine associated with restraint stress, suggesting an influential role of this cytokine in modulating intestinal permeability (Mazzon and Cuzzocrea 2008). Other studies have suggested that short‐term changes, such as high fat diets (Cani et al. 2008) and increased exercise (reviewed in De Oliveira et al. 2014) can greatly influence the permeability of the gut lining, though the precise mechanisms by which these effects take place are not completely understood.

Many diverse acute stressors (e.g., restraint, changes in diet, and increased exercise) can impact the vulnerability of the gut lining, suggesting that the microbiota within the gut may be changing as well. Whether an acute immune challenge influences the gut microbiome directly, however, and precisely how the gut epithelium responds, is not understood. The goal of this study was to determine how an immune challenge affects the gut microbiome of adult male and female Siberian hamsters and to shed light onto possible modulators of this response. Specifically, we hypothesized that if the gut axis interacts with the immune system during times of immune challenge, we would see alterations in gut microbial communities in LPS‐treated individuals, particularly those bacteria involved in reducing endotoxin levels (e.g., *Bifidobacteria*) (Cani et al. 2007a).

## Materials and Methods

### Ethical approval

All procedures were performed in accordance with the National Institutes of Health Guide for the Care and Use of Laboratory Animals and were approved by the Bloomington Institutional Animal Care and Use Committee at Indiana University (Protocol 16‐025). Investigators understand the ethical principles under which the journal operates, and all work complies with the journal's animal ethics checklist.

### Animals and housing conditions

Male and female adult hamsters (>60 days of age) bred in the laboratory were housed in a 16:8 light:dark photoperiod, in polypropylene cages (28 × 17 × 12 cm). Ambient temperature was maintained at 20 ± 2°C, and relative humidity was maintained at 55 ± 5%. Hamsters were given *ad libitum* access to tap water and standard laboratory rodent chow (Lab Diet 5001, PMI Nutrition). Males and females were run together in cohorts in the same animal holding room, housed individually for the duration of the study, and procedures were the same for all cohorts.

### Experiment 1: effects of LPS on cytokines and the HPA axis

#### Immune challenge

To determine the effects of LPS on the immune system and the HPA axis, in a subset of male and female hamsters, half of the animals received an intraperitoneal (i.p.) injection of LPS (25 *μ*g dissolved in 0.1 mL of 0.9% sterile saline; LPS from *Salmonella enterica* serotype typhimurium, Sigma‐Aldrich, St. Louis, MO, USA) (*n* = 36 males; *n* = 39 females), and the other half of the animals received a 0.1 mL injection of 0.9% sterile saline (*n* = 39 males; *n* = 37 females). At the end of each period (1, 2, 3 h postinjection), all animals were euthanized and organs were weighed.

#### Blood sampling and tissue collection

Because LPS stimulates an increase in cortisol that peaks around 2 h post‐injection (Bilbo et al. 2003; Owen‐Ashley et al. 2006), in the current study, we measured serum at 1, 2, and 3 h postinjection to capture the time point in which cortisol would likely be the highest. To do so, approximately half of the animals were lightly anesthetized with isoflurane vapors, and a baseline blood sample was collected from the retro‐orbital sinus prior to the animals receiving a single i.p. injection of LPS or saline as previously described. A second terminal blood sample was taken 1 h (*n* = 7 saline males, *n* = 6 LPS males; *n* = 6 saline females, *n* = 7 LPS females), 2 h (*n* = 7 saline males, *n* = 6 LPS males; *n* = 7 saline females, *n* = 7 LPS females), or 3 h (*n* = 7 saline males, *n* = 6 LPS males; *n* = 6 saline females, *n* = 7 LPS females) after injections for hormone analysis. Blood samples were allowed to clot at room temperature for 1 h, clots were removed, and samples were centrifuged at 4°C for 30 min at 1540 g. Serum was stored at −20°C until processed. Following the terminal blood samples, all animals were euthanized via a lethal i.p. injection of a ketamine and xylazine cocktail in 0.9% saline and livers were weighed.

In the other half of the animals, 1 h (*n* = 6 saline males, *n* = 5 LPS males; *n* = 5 saline females, *n* = 3 LPS females), 2 h (*n* = 5 saline males, *n* = 6 LPS males; *n* = 5 saline females, *n* = 4 LPS females), and 3 h (*n* = 5 saline males, *n* = 5 LPS males; *n* = 4 saline females, *n* = 5 LPS females) postinjection, animals were euthanized via an overdose of isoflurane followed by necropsy, and the liver was fast frozen and weighed for cytokine analysis.

#### Cortisol analysis

Serum cortisol concentrations in males and females were determined in multiple enzyme immunoassays (EIAs) from a commercially prepared kit (Cortisol EIA Kit; Enzo Life Sciences, Inc., Farmingdale, NY, USA) that was previously validated for use in Siberian hamsters (Carlton and Demas 2015). The assay is highly specific for cortisol, with corticosterone cross‐reactivity 27.7% and <4.0% for other steroid hormones. The sensitivity of the assay is 56.72 pg/mL. Samples were diluted 1:80 with assay buffer and run in duplicate (Carlton and Demas 2015; Rendon et al. 2015). Male and female samples were run on the same plates. The intra‐assay coefficient of variation was 6.71%, and the inter‐assay coefficient of variation was 5.86%.

#### Preparation of liver homogenate and TNF‐*α* analysis

To prepare liver tissue for cytokine analysis, 0.5 g samples of liver were homogenized on ice in 4.5 mL of phosphate buffer saline (PBS, pH 7.0, containing 0.25 mol/L sucrose) (Chang et al. 2013). Samples were then centrifuged at 3375 g for 1 h at 4°C and the supernatant was collected for further analysis. We measured TNF‐*α* levels in male and female liver tissue 1, 2, and 3 h postinjection using an enzyme‐linked immunosorbent assay (ELISA) from a commercially prepared kit validated before use on Siberian hamsters (TNF‐*α* ELISA Kit; Enzo Life Sciences, Inc., Farmingdale, NY, USA) (Shin et al. 2013; Yeon et al. 2015). The sensitivity of the assay is 3.9 pg/mL and is highly specific for bioactive TNF‐*α*. Samples were run neat and in duplicate. Nine samples from saline‐treated males and nine samples from saline‐treated females showed undetectable liver TNF‐*α* levels and were therefore assigned a value of 3.9 pg/mL for purposes of analysis. The intra‐assay coefficient of variation and inter‐assay coefficient of variation were 18.44% and 5.49%, respectively.

### Experiment 2: effects of LPS on the gut microbiome

#### Immune challenge and colonic temperature

To determine whether an immune challenge affected the microbial communities in the gut, male and female hamsters were assigned to an experimental group (*n* = 14 males; *n* = 10 females), in which they received a single i.p. injection of LPS, as previously described, or a control group (*n* = 14 males; *n* = 10 females), in which they received sterile saline on D8. During the study (days 1–7 [Pretreatment]; day 8 [Treatment]; days 9–21 [Posttreatment]), body mass, food intake, and colonic temperatures were measured at regular intervals in all animals. Additionally, following LPS or saline injections, behavior (attitude, posture, gait, movement) was also monitored to ensure the health of all animals. Colonic temperature was measured on days 1, 3, and 7 (Pretreatment), day 8 (Treatment), and days 9–14, 17, and 21 (Posttreatment) using a MicroTherma 2T thermometer (ThermoWorks, Alpine, UT, USA) and a lubricated RET‐3‐ISO thermocouple probe (Physitemp Instruments, Inc., Clifton, NJ, USA) inserted ∼12 mm into the rectum (Carlton and Demas 2015).

#### Fecal sampling and tissue collection

Animals were removed from their home cage and held over a sterile container to collect fecal samples, after which the samples were stored in −80°C until processed. All animals were returned to their home cage until the next assessment. At the end of the experiment (D21), all animals were euthanized via a lethal i.p. injection of a ketamine and xylazine cocktail in 0.9% saline and organs were weighed.

#### Microbiome analysis

DNA was extracted from fecal samples collected on day 7 (before treatment), day 10 (48 h posttreatment), and day 21 (end of experimentation) (*n* = 6 saline males, *n* = 6 LPS males, *n* = 4 saline females, *n* = 5 LPS females) using a commercially prepared kit (Promega's Maxwell^®^ RSC Tissue DNA Kit, Madison, WI). Before homogenizing samples, we added 300 *μ*L of lysis buffer provided in the kit to each sample and then centrifuged each sample at 4°C for 5 min at 355 g and used the supernatant for extraction.

We extracted three different negative control samples simultaneously while processing experimental samples for assessment of any background contamination (a: elution buffer only; b: sterile water + elution buffer; and c: TE buffer [supplied by Promega kit] + RNase A Solution [Promega, Madison, WI] + elution buffer).

We verified the purity of the DNA collected from each sample, and using Bioo Scientific's NEXTflex™ 16S V4 Amplicon‐Seq Library Prep Kit 2.0 (Austin, TX), multiplexed amplicon libraries spanning the V4 hypervariable domain of microbial 16S ribosomal RNA (rRNA) gene were prepared. Samples were cleaned using Agencourt AMPure XP Magnetic Beads, amplified using the supplied customized PCR primers that target the V4 domain, and sequence information was determined using the Illumina MiSeq v3 (600 cycle) platform in the Center for Genomics and Bioinformatics (CGB). Filtering, error correction, and removal of chimeras were completed, and sequences were then identified using Swarm and matched against the Silva database to identify operational taxonomic units (OTUs) (Mahé et al. 2014; Armanhi et al. 2016). For experimental samples, we found a mean of 60,344 sequences per sample. For negative control samples, we found a mean of 1 sequence per sample, suggesting little to no contamination of samples. The OTUs found in the negative controls were not found in experimental samples.

### Statistical analysis

All statistical analyses were performed in R v. 3.3.3 (R Core Team 2016). We attributed statistical significance at *P* < 0.05 and controlled for false discovery rate (FDR) when multiple comparisons were made. Data were checked for normality and homogeneity of variance, and data that could not be transformed to attain normality were analyzed using nonparametric tests. If a model reported a significant effect, two‐tailed *t tests* were run to determine pair‐wise relationships.

In Experiment 1, to test the effects of LPS treatment on body mass and organ mass, we used linear models (LMs) in which treatment (saline vs. LPS), hour (1, 2, 3 h postinjection), and their interactions were fixed effects. To analyze the effects of LPS treatment on cortisol, we used linear mixed models (LMMs) with “animal ID” as a repeated random factor and treatment (saline vs. LPS), time (pre‐ vs. postinjection), hour (1, 2, 3 h postinjection), sex (males vs. females), and their interactions as fixed effects (Rosvall 2013). We determined that there was a significant difference between cortisol in males and females (*F*_1,74_ = 40.073, *P* < 0.001); therefore all further analyses were completed on each sex independently. Furthermore, to analyze the effects of LPS treatment on liver TNF‐*α* levels, we used a LM in which treatment (saline vs. LPS), hour (1, 2, 3 h postinjection), sex (males vs. females), and their interactions were fixed effects. We determined that there was a significant difference between liver TNF‐*α* levels in males and females (*F*_1,46_ = 4.035, *P* = 0.050), therefore all further analyses were completed on males and females separately.

In Experiment 2, to compare body mass, food intake, and body temperature over time, we used repeated measures LMMs with “animal ID” as a random factor and treatment (saline vs. LPS), time (day), and their interactions as fixed effects. One saline‐treated female was excluded from analysis because she developed a skin wound and was treated by veterinary staff. To analyze organ mass in males and females, we used two‐tailed *t tests* to determine the effects of treatment on gross anatomy.

Two‐way analyses of variance (ANOVAs) were used to compare the effects of treatment (LPS and saline) and time (Pretreatment, Treatment, and Posttreatment) on bacterial phyla and families. Principle coordinate analysis (PCoA) was performed on the microbial communities to visualize differences between groups and time (Sze et al. 2014). To determine alpha diversity, we calculated the Shannon–Wiener index and ran two‐way analyses of variance (ANOVAs) to determine statistically significant changes in the alpha diversity (Hill 1973; Jost 2006). Furthermore, to determine beta diversity, we calculated Bray–Curtis dissimilarity scores across groups and time points. A Bray–Curtis dissimilarity score of zero represents groups that are similar in composition, and a score of 1 represents groups that do not share microbial composition. We also converted Bray–Curtis dissimilarity scores to percent differences between groups for a more clear comparison. Finally, to determine if bacterial communities were affected by treatment (LPS vs. saline), time (pretreatment, treatment, or posttreatment), or the interaction between treatment and time, we used multivariate nonparametric ANOVA of dissimilarities (PERMANOVA) with 999 permutations, using the Adonis function with the Hellinger transformation in the Vegan package in R (Oksanen et al. 2007; Oksanen 2015; Schriever and Lytle 2016) based on Euclidean distance.

## Results

### Experiment 1

#### LPS treatment did not affect body mass or liver mass within 3 h of injection

At the start of the experiment, female body mass did not significantly differ across groups (treatment: *F*_1,70_ = 1.197, *P* = 0.281; time of bleed: *F*_3,70_ = 0.282, *P* = 0.756), and male body mass did not significantly differ across groups (treatment: *F*_1,78_ = 2.013, *P* = 0.160; time of bleed: *F*_3,70_ = 0.556, *P* = 0.646). As predicted, within the short time frame in the first experiment, LPS treatment did not affect body mass in females (*F*_1,72_ = 0.138, *P* = 0.712) or males (*F*_1,79_ = 2.023, *P* = 0.159). The hour postinjection also did not have a significant effect on body mass in females (*F*_1,72_ = 0.040, *P* = 0.960) or males (*F*_2,79_ = 0.526, *P* = 0.593). Furthermore, liver mass was not affected by LPS treatment or hour postinjection. Specifically, in females, LPS treatment (*F*_1,72_ = 1.284, *P* = 0.261) and the hour postinjection (*F*_2,72_ = 0.057, *P* = 0.945) did not affect liver mass. Similarly, in males, LPS treatment (*F*_1,79_ = 0.093, *P* = 0.761) and the hour postinjection (*F*_2,79_ = 1.004, *P* = 0.371) did not affect liver mass (Table 1).

**Table 1:** Experiment 1: Means ± SEM of body and liver masses across treatment groups and sexes

	Body mass (g)		Liver mass (g)	
	Male	Female	Male	Female
Saline	47.410 ± 0.916	42.000 ± 1.251	1.870 ± 0.044	1.766 ± 0.078
LPS	49.288 ± 0.939	41.313 ± 2.980	1.850 ± 0.043	1.652 ± 0.062

No values were significantly different across treatment groups.

#### LPS treatment elevated serum cortisol

**Figure 1:**
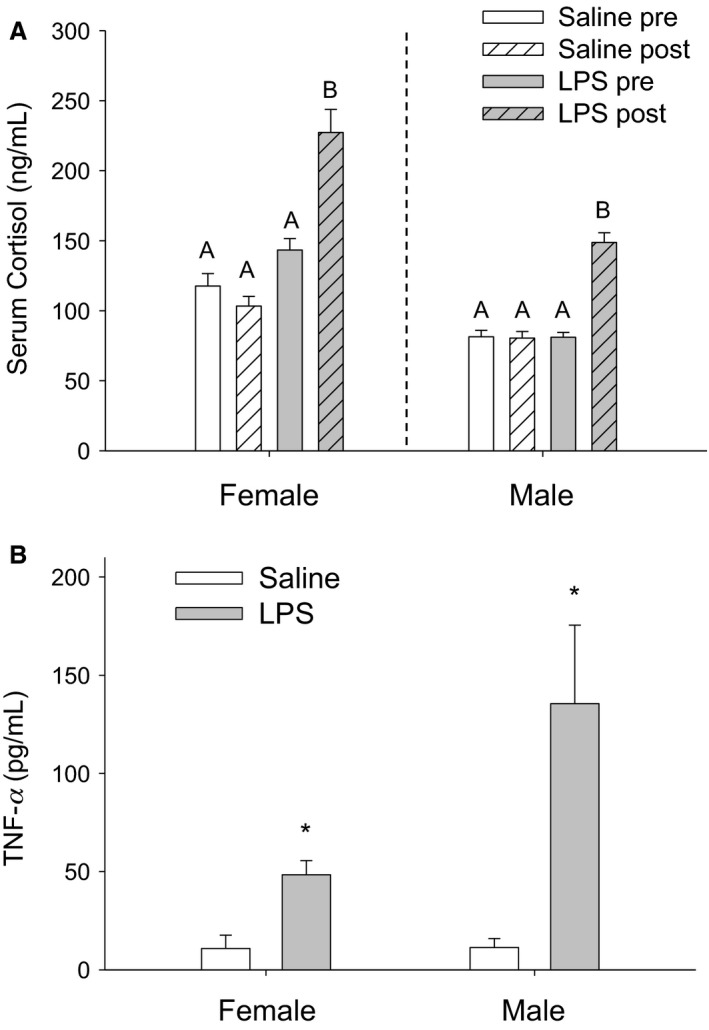
Effects of LPS treatment on cortisol (A) and liver TNF‐*α* (B) in female and male hamsters. Mean ± SEM circulating serum cortisol following saline and LPS treatment in female hamsters and male hamsters (A). The three time points (1, 2, and 3 h posttreatment) were combined here, as there were no significant differences across time points). Groups within each sex with different letters indicate statistically significant differences between group means (*P *< 0.05); groups sharing the same letter within each sex are statistically equivalent. Mean ± SEM liver TNF‐*α* levels in females and males (B) combined across time points. White bars represent saline‐treated animals, and grey bars represent LPS‐treated animals. An asterisk (*) indicates statistically significant differences between group means within each sex at *P* < 0.05.

LPS treatment significantly increased cortisol levels in females (*F*_1,68_ = 42.181, *P* < 0.001) and males (*F*_1,66_ = 49.508, *P* < 0.001) postinjection when compared with cortisol levels in saline‐treated animals (Fig. 1A). The hour postinjection, however, did not significantly affect the increase in cortisol levels in females (*F*_2,68_ = 1.112, *P* = 0.335) or males (*F*_2,66_ = 0.358, *P* = 0.700).

#### LPS treatment increased liver TNF‐*α*

LPS treatment affected liver TNF‐*α* levels in females (*F*_1,20_ = 13.867, *P* = 0.001; Fig. 1B) and males (*F*_1,26_ = 11.166, *P* = 0.003; Fig. 1B). In particular, LPS treatment increased liver TNF‐*α* levels in both sexes when compared with liver TNF‐*α* levels in saline‐treated animals. The hour postinjection, however, did not significantly affect the increase in liver cytokine levels in females (*F*_2,20_ = 1.246, *P* = 0.309) or males (*F*_2,26_ = 1.315, *P* = 0.286).

### Experiment 2

#### LPS treatment decreased body mass

**Figure 2:**
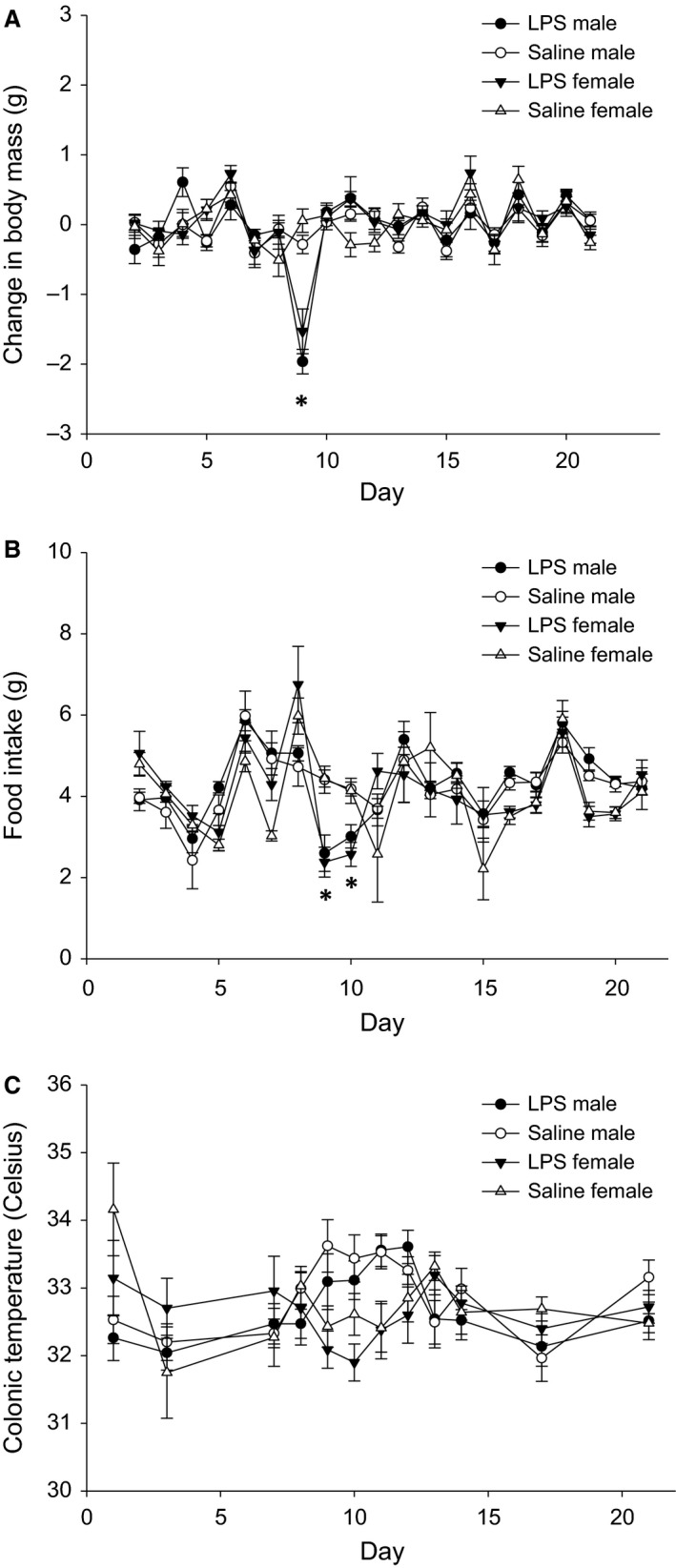
Mean ± SEM of (A) body mass, (B) food intake, and (C) colonic temperature over time in female and male hamsters. White circles represent saline−treated males; white triangles represent saline−treated females; black circles represent LPS‐treated males; and black triangles represent LPS‐treated females. An asterisk (*) indicates statistically significant differences between group means within each sex at *P* < 0.05.

At the start of the second 21‐day experiment, female body mass was not significantly different across treatment groups (*F*_1,18_ = 0.698, *P* = 0.414). However, body mass decreased in LPS‐treated females [day x treatment interaction (*F*_19,380_ = 3.995, *P* < 0.001)]. Specifically, body mass was significantly lower in LPS‐treated females on day 9 (*P* = 0.024; 24 h post‐LPS injections), with all other days not significantly different between treatment groups (*P* > 0.05 in all cases) (Fig. 2A).

Similarly, in males, at the start of the second experiment, body mass was not significantly different across treatment groups (*F*_1,26_ = 1.422, *P* = 0.244). However, body mass decreased in LPS‐treated animals [day × treatment interaction (*F*_19,560_ = 4.532, *P* < 0.001)]. Specifically, body mass was significantly lower in LPS‐treated males on day 9 (*P* = 0.020), with all other days not significantly different between treatment groups (*P* > 0.05 in all cases) (Fig. 2A).

#### LPS treatment reduced food intake

In females, food intake was significantly decreased in LPS‐treated females [day x treatment interaction (*F*_19,346.92_ = 2.350 *P* = 0.001)]. Specifically, food intake was significantly lower in LPS‐treated females on days 9 (*P* = 0.008) and 10 (*P* = 0.020), with all other days not significantly different between treatment groups (*P* > 0.05 in all cases) (Fig. 2B).

Similarly, in males, food intake was significantly decreased in LPS‐treated animals [day x treatment interaction (*F*_19,530.19_ = 1.985, *P* = 0.008)]. Specifically, food intake was significantly lower in LPS‐treated males on days 9 (*P* = 0.034) and 10 (*P* = 0.034), with all other days not significantly different between treatment groups (*P* > 0.05 in all cases) (Fig. 2B).

#### LPS treatment did not affect body temperature

In females, LPS treatment (*F*_1,20_ = 1.036, *P* = 0.321) and the treatment x day interaction (*F*_11,220_ = 1.318, *P* = 0.216) had no significant effect on colonic temperature. LPS‐treated females, however, showed hypothermia on days 9 and 10 (24–48 h post‐LPS injection), albeit not significant (Fig. 2C). There was a significant effect of day on colonic temperature in both treatment groups (*F*_11,220_ = 2.862, *P* = 0.006).

Similarly, in males, LPS treatment (*F*_1336_ = 2.327, *P* = 0.128) and the treatment x day interaction (*F*_11,336_ = 0.636, *P* = 0.798) had no significant effect on colonic temperature in males. LPS‐treated males, however, showed hypothermia on days 9 and 10, though this decrease in temperature was not significant (Fig. 2C). There was a significant effect of day on colonic temperature in both treatment groups (*F*_11,336_ = 6.366, *P* < 0.001).

#### LPS treatment did not affect organ mass

LPS treatment did not affect organ mass in females or males at the end of 21 days. Specifically, in females, there was no effect of LPS treatment on liver mass (*F*_1,17_ = 1.501e‐05, *P* = 0.997), spleen mass (*F*_1,17_ = 0.074, *P* = 0.789), ovarian mass (*F*_1,17_ = 0.022, *P* = 0.884), or uterine horn mass (*F*_1,17_ = 0.042, *P* = 0.841). Similarly, in males, there was no effect of LPS treatment on liver mass (*F*_1,26_ = 0.675, *P* = 0.419), spleen mass (*F*_1,26_ = 1.490, *P* = 0.233), or paired testes mass (*F*_1,26_ = 0.127, *P* = 0.724) (Table 2).

**Table 2:** Experiment 2: Means ± SEM of body and organ masses across treatment groups and sexes

	Saline		LPS	
	Male	Female	Male	Female
Liver mass (g)	1.816 ± 0.094	1.905 ± 0.132	1.937 ± 0.114	1.823 ± 0.156
Spleen mass (g)	0.067 ± 0.005	0.074 ± 0.007	0.081 ± 0.010	0.078 ± 0.009
Ovarian or testes mass (g)	0.697 ± 0.03	0.010 ± 0.001	0.714 ± 0.034	0.010 ± 0.001
Uterine horn mass (g)		0.132 + 0.021		0.126 + 0.009

No values were significantly different across treatment groups.

#### LPS treatment did not affect microbial community composition

**Figure 3:**
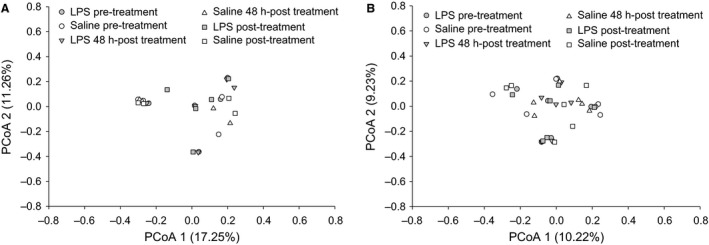
Principle Coordinates Analysis (PCoA) analysis of female (A) and male (B) hamsters, PERMANOVA, *P* > 0.05. White circles represent the Pre−treatment time point in saline‐treated hamsters; white triangles represent 48 h post‐injection in saline−treated hamsters; white squares represent the Post−treatment time point in saline‐treated hamsters; gray circles represent the Pretreatment time point in LPS‐treated hamsters; gray triangles represent 48 h post−injection in LPS−treated hamsters; and gray squares represent the Post−treatment time point in LPS‐treated hamsters.

Gut microbial communities in females and males were significantly different from each other (*F*_1,62_ = 6.958, *P* = 0.004), therefore all further analyses on the gut microbiome were completed on each sex independently. In males and females gut bacterial community composition did not significantly differ across treatments or time. Specifically, in females, treatment (LPS vs. saline) (*F*_1,26_ = 0.702, *P* = 0.522), time (Pretreatment, Treatment, Posttreatment) (*F*_2,26_ = 0.021, *P* = 0.995), and the interaction of treatment × time (*F*_2,26_ = 0.015, *P* = 1.000) had no significant effect on bacterial communities. Similarly, in males, treatment (LPS vs. saline) (*F*_1,35_ = 1.498, *P* = 0.230), time (Pretreatment, Treatment, Posttreatment) (*F*_2,35_ = 0.044, *P* = 0.997), and the interaction of treatment x time (*F*_2,35_ = 0.0384, *P* = 0.996) had no significant effect on bacterial communities. The relative abundance of OTUs in females and males is plotted in the Principle Coordinates Analysis (PCoA) plots in Figure 3A and B, respectively, showing that the bacterial communities across treatment groups and time within each sex were very similar to one another.

#### LPS treatment did not affect microbial diversity

**Figure 4:**
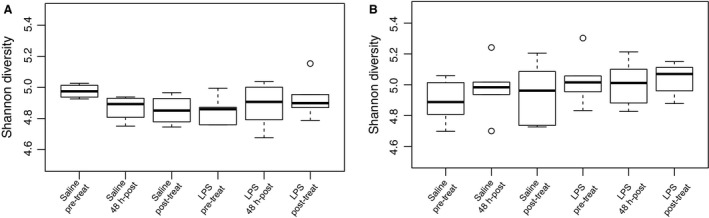
Shannon−Wiener diversity across treatment groups and time in female (A) and male (B) hamsters. Shannon diversity did not significantly differ across time points or treatment in females or males (*P* > 0.05 in all cases).

In females, Shannon diversity across treatment groups and time did not significantly differ (*F*_5,21_ = 0.878, *P* = 0.513). Shannon–Wiener index before treatment was 4.976 in control animals and 4.850 in LPS animals. 48 h after treatment, the Shannon–Wiener index was 4.869 in control animals, and 4.883 in LPS animals. Following the recovery period, the Shannon–Wiener index was 4.853 in control animals 4.933 in LPS animals (Table 3, Fig. 4).

**Table 3:** Experiment 2: Measures of alpha diversity in male and female hamsters across treatment groups and time

	Saline			LPS		
	Pre	Treat	Post	Pre	Treat	Post
Female						
Shannon index	4.976	4.869	4.853	4.850	4.883	4.933
Male						
Shannon index	4.892	4.977	4.946	5.030	5.007	5.040

Similarly, in males, Shannon diversity across treatment groups and time did not significantly differ (*F*_5,30_ = 0.787, *P* = 0.567). The Shannon–Wiener index before treatment was 4.892 in control animals and 5.030 in LPS animals. 48 h after treatment, the Shannon–Wiener index was 4.977 in control animals, and in LPS animals, the Shannon–Wiener index was 5.007. Following the recovery period, the Shannon–Wiener index was 4.946 in control animals and 5.040 in LPS animals (Table 3, Fig. 4).

Furthermore, according to the Bray–Curtis dissimilarity scores, before treatment, LPS‐treated females were 15.177% different from saline‐treated females. 48 h after treatment, LPS‐ and saline‐treated females were 17.384% different from each other, and following the recovery period, the groups were 12.899% different from each other. Within treatment group dissimilarity comparisons are shown in Table 4.

**Table 4:** Experiment 2: Female Bray–Curtis dissimilarity scores across groups and time points

	Saline pre	Saline treat	Saline post	LPS pre	LPS treat
Saline treat	0.085				
Saline post	0.164	0.138			
LPS pre	0.152	0.139	0.138		
LPS treat	0.192	0.174	0.121	0.101	
LPS post	0.197	0.194	0.129	0.115	0.079

Similarly, before treatment, LPS‐treated males were 19.062% different from saline‐treated males. 48 h after treatment, LPS‐ and saline‐treated males were 13.529% different from each other, and following the recovery period, the groups were 11.902% different from each other. Within treatment group dissimilarity comparisons are shown in Table 5.

**Table 5:** Experiment 2: Male Bray–Curtis dissimilarity scores across groups and time points

	Saline pre	Saline treat	Saline post	LPS pre	LPS treat
Saline treat	0.113				
Saline post	0.137	0.100			
LPS pre	0.191	0.142	0.128		
LPS treat	0.191	0.135	0.123	0.087	
LPS post	0.175	0.131	0.119	0.076	0.085

#### LPS treatment did not affect phyla or families in the gut microbiome

In females and males, there were no significant effects of treatment, time, or the treatment × time interaction for all 14 phyla present in the gut microbiome (*P* > 0.05 in all cases; Table 6), nor were there any effects of treatment, time, or the treatment x time interaction for all 60 families of bacteria in the gut (*P* > 0.05 in all cases).

**Table 6:** Experiment 2: Effects of LPS on the relative abundance of bacterial phyla in female and male hamsters. No values were significantly different

**Phylum**	**Parameter**	**Female**	**Male**
Actinobacteria	Treatment	*F*_1,10_ = 1.983, *P* = 0.742	*F*_1,7_ = 0.057, *P* = 0.976
Actinobacteria	Time	*F*_2,20_ = 1.066, *P* = 0.742	*F*_2,14_ = 0.847, *P* = 0.858
Actinobacteria	Treatment × time	*F*_2,20_ = 0.409, *P* = 0.855	*F*_2,14_ = 1.996, *P* = 0.732
Bacteroidetes	Treatment	*F*_1,10_ = 2.675, *P* = 0.742	*F*_1,7_ = 0.192, *P* = 0.927
Bacteroidetes	Time	*F*_2,20_ = 0.375, *P* = 0.855	*F*_2,14_ = 0.744, *P* = 0.863
Bacteroidetes	Treatment × time	*F*_2,20_ = 0.486, *P* = 0.855	*F*_2,14_ = 0.392, *P* = 0.927
Cyanobacteria	Treatment	*F*_1,10_ = 0.241, *P* = 0.855	*F*_1,7_ = 0.056, *P* = 0.976
Cyanobacteria	Time	*F*_2,20_ = 1.012, *P* = 0.742	*F*_2,14_ = 0.025, *P* = 0.976
Cyanobacteria	Treatment × time	*F*_2,20_ = 0.186, *P* = 0.917	*F*_2,14_ = 1.365, *P* = 0.732
Deferribacteres	Treatment	*F*_1,10_ = 0.752, *P* = 0.742	*F*_1,7_ = 1.178, *P* = 0.732
Deferribacteres	Time	*F*_2,10_ = 1.237, *P* = 0.742	*F*_2,14_ = 1.707, *P* = 0.732
Deferribacteres	Treatment × time	*F*_2,20_ = 0.686, *P* = 0.855	*F*_2,14_ = 1.064, *P* = 0.771
Elusimicrobia	Treatment	*F*_1,10_ = 1.778, *P* = 0.742	*F*_1,7_ = 0.001, *P* = 0.976
Elusimicrobia	Time	*F*_2,20_ = 0.157, *P* = 0.917	*F*_2,14_ = 3.083, *P* = 0.732
Elusimicrobia	Treatment × time	*F*_2,20_ = 1.957, *P* = 0.742	*F*_2,14_ = 0.337, *P* = 0.927
Euryarchaeota	Treatment	*F*_1,10_ = 0.327, *P* = 0.855	*F*_1,7_ = 0.417, *P* = 0.871
Euryarchaeota	Time	*F*_2,20_ = 2.335, *P* = 0.742	*F*_2,14_ = 1.891, *P* = 0.732
Euryarchaeota	Treatment × time	*F*_2,20_ = 2.018, *P* = 0.742	*F*_2,14_ = 0.766, *P* = 0.863
Firmicutes	Treatment	*F*_1,10_ = 3.019, *P* = 0.742	*F*_1,7_ = 0.131, *P* = 0.927
Firmicutes	Time	*F*_2,20_ = 0.413, *P* = 0.855	*F*_2,14_ = 0.579, *P* = 0.891
Firmicutes	Treatment × time	*F*_2,20_ = 0.556, *P* = 0.855	*F*_2,14_ = 0.358, *P* = 0.927
Fusobacteria	Treatment	*F*_1,10_ = 1.00, *P* = 0.742	*F*_1,7_ = 0.032, *P* = 0.976
Fusobacteria	Time	*F*_2,20_ = 1.00, *P* = 0.742	*F*_2,14_ = 0.502, *P* = 0.923
Fusobacteria	Treatment × time	*F*_2,20_ = 1.00, *P* = 0.742	*F*_2,14_ = 1.538, *P* = 0.732
Proteobacteria	Treatment	*F*_1,10_ = 0.360, *P* = 0.855	*F*_1,7_ = 1.413, *P* = 0.732
Proteobacteria	Time	*F*_2,20_ = 0.213, *P* = 0.917	*F*_2,14_ = 2.101, *P* = 0.732
Proteobacteria	Treatment × time	*F*_2,20_ = 0.402, *P* = 0.855	*F*_2,14_ = 1.324, *P* = 0.732
Saccharibacteria	Treatment	*F*_1,10_ = 0.511, *P* = 0.855	*F*_1,7_ = 0.002, *P* = 0.976
Saccharibacteria	Time	*F*_2,20_ = 2.761, *P* = 0.742	*F*_2,14_ = 1.181, *P* = 0.743
Saccharibacteria	Treatment × time	*F*_2,20_ = 4.181, *P* = 0.638	*F*_2,14_ = 3.939, *P* = 0.732
Spirochaetae	Treatment	*F*_1,10_ = 0.027, *P* = 0.917	*F*_1,7_ = 0.418, *P* = 0.871
Spirochaetae	Time	*F*_2,20_ = 1.384, *P* = 0.742	*F*_2,14_ = 2.948, *P* = 0.732
Spirochaetae	Treatment × time	*F*_2,20_ = 0.099, *P* = 0.928	*F*_2,14_ = 1.944, *P* = 0.732
Tenericutes	Treatment	*F*_1,10_ = 1.912, *P* = 0.742	*F*_1,7_ = 0.005, *P* = 0.976
Tenericutes	Time	*F*_2,20_ = 2.944, *P* = 0.742	*F*_2,14_ = 1.623, *P* = 0.732
Tenericutes	Treatment × time	*F*_2,20_ = 1.275, *P* = 0.742	*F*_2,14_ = 0.067, *P* = 0.976
Verrucomicrobia	Treatment	*F*_1,10_ = 0.053, *P* = 0.917	*F*_1,7_ = 2.360, *P* = 0.732
Verrucomicrobia	Time	*F*_2,20_ = 5.144, *P* = 0.638	*F*_2,14_ = 2.672, *P* = 0.732
Verrucomicrobia	Treatment × time	*F*_2,20_ = 0.166, *P* = 0.917	*F*_2,14_ = 1.539, *P* = 0.732
Other	Treatment	*F*_1,10_ = 0.008, *P* = 0.931	*F*_1,7_ = 0.044, *P* = 0.976
Other	Time	*F*_2,20_ = 1.549, *P* = 0.742	*F*_2,14_ = 1.022, *P* = 0.771
Other	Treatment × time	*F*_2,20_ = 0.987, *P* = 0.742	*F*_2,14_ = 1.557, *P* = 0.732

## Discussion

Activation of the immune system may influence the vulnerability of the gut to pathogenic bacteria; how the immune system communicates with the gut microbiome and the time course of this communication, however, is not completely understood. Exogenous LPS produces a rapid and robust activation of the immune system, though whether an acute immune challenge influences the gut microbiome directly has not been thoroughly investigated. In this study, we tested the effects of an immune challenge on the gut microbiome at the conclusion of the sickness response (48 h after challenge) by administering exogenous LPS and evaluating measures of immunity and changes in the gut microbiome. Because other acute stressors, such as exercise and short‐term changes in diet can influence the gut microbiome, it seemed that a single, but robust immune challenge would likely be associated with similar changes in these communities.

We determined, however, that the dose of LPS was effective in immediately increasing levels of glucocorticoids and at least one proinflammatory cytokine in the liver particularly important in gut permeability, TNF‐*α*, but this same immune challenge was not associated with changes in the gut microbiota 48 h after. Previous work has suggested that serum cytokine levels (e.g., IL‐1*α*, IL‐1*β*, IL‐10) stay increased for up to 4 h after LPS injection, but that TNF‐*α* may remain elevated in the brain for up to 28 h post‐LPS injection (Erickson and Banks 2011), suggesting that the cytokine may still be capable of influencing physiology even after the initial immune response. It is possible, however, that 48 h post‐LPS injection, the microbiome may have recovered back to its normal state, as body mass, but not food intake, had done. It seems more likely, however, that healthy individuals may have the necessary homeostatic mechanisms to prevent the actions of LPS from producing long‐term gut dysbiosis. These data suggest the downstream effects of this type of immune activation may be transient. Furthermore research should investigate whether an acute immune activation affects the gut microbiome more quickly via monitoring changes in the microbiome immediately after the injection. Additionally, because the immune system and the microbiome are fully developed by adulthood, it is possible that an immune activation may affect an individual's microbiome more strongly during early development, when both the microbiome and the immune system are developing in parallel.

Because food intake was still significantly different at day 10 in both sexes, we would have predicted to see changes in the gut microbiome, as reduced food intake (and often decreased peristalsis) is often associated with changes in the microbiota (Kim et al. 2016). The lack of change in the microbiome that we found may suggest that in the face of these acute stressors (immune challenge, changes in food intake), a healthy microbiome may be able to adjust to these changes so as to maintain homeostasis. What we do not know, however, is whether the gut lining was affected by this acute challenge, and further research should investigate this matter.

Moreover, one of the more important aspects of the LPS‐mediated immune response involves activation of TLR‐4, which increases circulating glucocorticoids and proinflammatory cytokines and contributes to the sickness response seen here and elsewhere (Bilbo and Schwarz 2012; Harvey and Boksa 2012; French et al. 2013). The upregulation of TRL‐4 in response to an immune activation, however, is short‐term, acting only to elicit appropriate physiological and behavioral responses to the acute immune challenge. The acute sickness response (e.g., lethargy, decreased food intake) enables healthy individuals to save energy needed to recover from infection. Long‐term upregulation of TRL‐4 may be an important contributor to gut dysbiosis, where the increase in TRL‐4 stimulates a lasting, low‐dose immune response without proper mechanisms to prevent dysfunction. This is present, for example, in patients with inflammatory bowel disease that have unregulated TLR‐4 expression in the mucosa of the intestines and changes in their gut microbial communities. This may suggest that abnormal TLR‐4 expression may play an important role in the loss of tolerance to enteric bacteria (Cario and Podolsky 2000), leading to endotoxin transfer from inside the gut to outside, where levels are elevated for long periods of time.

While it was hypothesized that an immune challenge would have effects on the microbiome either directly by the immune system or by way of the HPA axis, it appears that an upregulation of HPA activity itself due to a behavioral challenge, may more strongly influence microbial communities. For example, humans who respond to a single, high‐stress situation with an increased cortisol response also exhibit increased intestinal permeability (Vanuytsel et al. 2014). These findings suggest that cortisol and its precursors may act as important direct modulators of the microbiota, rather than indirectly being activated by the immune system. Therefore, the lack of gut dysbiosis in our study 48 h post‐LPS injection may possibly be explained by the fact that HPA activity in this case is mediated by the immune system and not by the stressor itself. Further work will investigate precisely how interactions among the immune system, the HPA axis, and the gut microbiome occur. Moreover, the gut lining may play a particularly important role in maintaining homeostasis in the body, and therefore, studies investigating the mechanisms that modulate how the tight junctions in the gut epithelium are maintained are particularly important.

Though we predicted a change in the microbiome in response to LPS similar to other acute stressors (e.g., restraint stress), we suggest that there are mechanisms to prevent gut dysbiosis in the face of an acute immune challenge. Furthermore, we provide evidence that the microbiome in healthy individuals does not fluctuate despite the effort needed to overcome other aspects of the sickness response. Collectively, these data suggest that an acute immune activation may not be capable of significantly altering the gut microbiome in healthy individuals. Investigations into precisely how an acute immune challenge influences the gut lining and whether there are more subtle shifts in the microbiome immediately following this stressor, however, are still needed. More broadly, the results of this study provide evidence that the immune system works in more indirect ways to influence the gut microbiome long‐term. The basic understanding that we provide here of how these types of acute stressors affect the microbiome offers important insight into the intricate relationships among the microbiome, the immune system, and the central nervous system, and it opens the door to future research investigating these connections.

## Conflict of Interest

The authors declare no conflict of interest, financial or otherwise.

## Research Governance

Fred H. Cate, Vice President for Research (vpr@iu.edu).
